# 

**Published:** 1882-07-20

**Authors:** 


**PLEASE NOTICE.**—Subscribers in arrears please remit without further delay.

SUBSCRIBERS Receipted will appear in our next.

SEE the advertisements of two Medical Colleges in this Journal.

THE next meeting of the American Medical Association is appointed for Cleveland, Ohio, June, 1883.

CORRECTION.—In Dr. Hobbs' article in the June number of our Journal on *Entropion and Trichiasis* in 23d line from the end read eliptical for conical, and in 17th and 19th lines from the end 10 and ten should read — 10.

SOUTHERN MEDICAL COLLEGE, ATLANTA.—The curriculum in this new and progressive Institution is now complete—the following additional lecturers having been appointed:

H. F. Scott, M. D., Lecturer on Dermatology.

B. H. Catching, D. D. S., Lecturer on Dental Surgery.

W. C. Jarnagin, M. D., Auxiliary to the chair of Obstetrics and Diseases of Women and Children.

